# Antioxidative and Immunomodulating Properties of *Aronia melanocarpa* Extract Rich in Anthocyanins

**DOI:** 10.3390/plants11233333

**Published:** 2022-12-01

**Authors:** Kseniya Bushmeleva, Alexandra Vyshtakalyuk, Dmitriy Terenzhev, Timur Belov, Evgeny Nikitin, Vladimir Zobov

**Affiliations:** A.E. Arbuzov Institute of Organic and Physical Chemistry, Kazan Scientific Center, Russian Academy of Sciences, Arbuzov Str. 8, Kazan 420088, Russia

**Keywords:** fruits of *Aronia melanocarpa*, bioactive compounds, extraction, anthocyanins, antioxidant capacity, immunomodulatory activity, oxidative stress damage

## Abstract

The fruits of *Aronia melanocarpa* are well known due to their high anthocyanin content that may be effective in preventing certain health disorders arising from oxidative stress. Various polyphenolic compounds such as anthocyanins and flavonoids are responsible for the multiple effects of chokeberry. The aim of this study was to determine in vitro how active the black chokeberry anthocyanins are in scavenging radicals and to evaluate in vivo their immunomodulating capacity. Using the method of column chromatography, we extracted the anthocyanins of black chokeberries, i.e., cyanidin-3-O-galactoside with a purity of over 93.7%. Using HPLC and spectrophotometric analysis, the flavonoid content was determined. Following the analysis of the tests with AAPH and DPPH, the chokeberry cyanidin-3-O-galactoside was found much better than individual anthocyanins in regard to antioxidant capacity. The range of concentrations was revealed, showing the protective effect of anthocyanins on the RPMI-1788 cell culture against cyclophosphamide, as well as against osmotic and peroxide hemolysis. An immunomodulating effect on the functional activity of phagocytes was revealed in vivo as a result of oral administration of chokeberry cyanidin-3-O-galactoside and a mixture composed of cyanidin-3-O-glucoside and cyanidin-3-O-galactoside standards. Consequently, anthocyanins, in particular cyanidin-3-O-galactoside, play an important role, demonstrating immunomodulating effects when chokeberries are consumed.

## 1. Introduction

Aronia (black chokeberry *or Aronia melanocarpa, Rosaceae*) as a plant has become very popular recently as its berries are rich in polyphenolic compounds in general and in anthocyanins specifically [1]. Proanthocyanidins (oligomeric and polymeric (epi)catechins) are the dominant phenolic compounds in chokeberry fruits, with their content reaching 5% of the whole fruit dry mass [2,3]. Anthocyanins represent about 25% of total chokeberry phenols and include cyanidin 3-glucoside, 3-galactoside, 3-xyloside, 3-arabinoside, pelargonidin-3-galactoside and pelargonidin-3-arabinoside [3,4,5]. The berries of a chokeberry possess high antioxidant potential, usually higher than that of the other tested plant materials [6]. The antioxidant potential of black chokeberry fruit and products is mainly attributed to polyphenols and was confirmed in various assays [4]. In fact, as shown by the authors of [7,8] based on the analysis of the ability to scavenge oxygen radicals, they have the highest antioxidant activity among the berries and other fruits studied up to now.

Anthocyanins are flavonoid compounds formed by a combination of anthocyanins and sugars. Plant anthocyanins have apparent antioxidant [9,10] and anti-inflammatory [11,12] properties. According to the existing literature, anthocyanins can be used as functional active ingredients and therapeutic agents for the prevention and treatment of diseases [13,14]. The prerequisite for their practical application is the effective extraction and purification of anthocyanins. The appropriate identification and quantification of anthocyanins and their derivatives are important to determine the contribution of food compounds to health because slight structural variations may alter gastrointestinal stability, bioavailability and hence the effect on the whole body [15].

In a previous study, we reported the method of preparation, extraction and characterization and the antioxidant and immunomodulating activity of *Aronia melanocarpa* berry extracts [16]. However, knowledge of the extract’s chemical composition is a prerequisite for the study of its potential mechanisms as well as the correlation between the content of bioactive substances and their activity [17]. Therefore, this study focused on the purification, characteristics and immunomodulating activity of anthocyanin-enriched extracts of *Aronia melanocarpa*. The data given by us in this work in regard to purification, characteristics and immunomodulating activity of the Aronia anthocyanin extract have not been previously found in literary sources. Thus, we have suggested that Aronia fruit extract, rich in anthocyanins, can be an effective antioxidant and immunomodulator to reduce the effects of stress and increase the body’s barrier functions.

Therefore, the aim of this study was to develop a more efficient method for isolating individual cyanidins with a high degree of purity and to evaluate the contribution of the Aronia anthocyanin extracts to the antioxidative and immunomodulating activity based on which one can evaluate its applicability as an immunomodulator. In addition, purified cyanidins and well-known immunomodulators such as *Echinacea purpurea* herb tincture were tested for comparison.

## 2. Results

### 2.1. Chemical Composition and Antioxidant Activity of Extracts

The total contents of sugars and natural antioxidants are given in Table 1. The content of anthocyanins in the original 70% ethanol extract of Aronia was high, which was consistent with other studies [18]. The cyanidin-3-O-galactoside fraction of Aronia extract (Cy-3-Gal Aronia) obtained after purification with NKA-9 resin (macroporous adsorption resin) had a low sugar content relative to Aronia 70% ethanol extract (sugar content decreased by 99.8%), with anthocyanins increasing from 93.6 mg/g to 917.31 mg/g (on dry basis). It had also almost half the number of total flavonoids, which are also responsible for antioxidant properties.

For the two extracts of *A. melanocarpa*, the main anthocyanins were identified by HPLC-MS based on their retention time in the chromatographic column, elution order, spectroscopic characteristics and fragmentation pattern. The results of the analysis are given in Table 2. Four major anthocyanins peaks were identified, consisting of cyanidin-3-O-galactoside, cyanidin-3-O-glucoside, cyanidin-3-O-arabinozide and cyanidin-3-O-xyloside. The amount of anthocyanins identified relative to their total content was 98.52% for Aronia 70% ethanol extract and 97.5% for anthocyanin fraction (Cy-3-Gal Aronia).

As shown in Table 2, the major compound in both extracts was cyanidin-3-O-galactoside (Cy-3-Gal). Its content in the anthocyanin fraction after column chromatography increased from 58.97 mg/g to 735.33 mg/g (on dry extract basis). The content of Cy-3-Gal in Cy-3-Gal Aronia compared to the remaining anthocyanins was 82.2%, which corresponded to the results of similar work, namely 82.5% according to [19].

Since Cy-3-Gal was the main compound in the anthocyanin fraction and accounted for 73.53% of the total mass of the extract, we assume that the underlying mechanism of antioxidant action is based on it. Therefore, a parallel work was carried out to evaluate the antioxidant action of the isolated and purified fraction, mainly consisting of high-purity Cy-3-Gal (Table 3, Fraction 6–9).

The method of column chromatography identified four groups of fractions consisting mainly of high-purity (from 93.7 to 97.8%) anthocyanins, the remaining fractions were of no interest as they were the mixtures containing anthocyanins in low concentrations (Figure S2). These individual fractions were identified using hydrophilic HPLC with a reversed-phase column, and their percentages relative to other components are presented in Table 3.

Among the compounds identified by the HPLC-MS method, we also determined the flavonol quantity in 70% ethanol extract and anthocyanin extract of *A. melanocarpa*, belonging to the flavonoid class in the individual and glycoside forms, as shown in Table 4. Note that this class of compounds also contributed to the overall antioxidant activity.

The detected flavonol content for ethanol extract was 3.35% on a dry extract basis and 89.2% of the total flavonoids; in the case of the anthocyanin fraction of Cy-3-Gal Aronia, it was 1.38% on a dry extract basis and 89.69% of the total flavonoids. The remaining compounds of the dry extract belonged to the class of flavone acids and tannins. In the anthocyanin Cy-3-Gal Aronia extract, these compounds were below the detection limit.

The electrospray ionization (ESI) mass spectra were taken in the negative scanning mode with the formation of a deprotonated [M-H] ion, where H = 1 (proton mass). Mass fragmentation of the peak of fractions 6–9 and 13–15 revealed two main fragments: M—1 = 447 and 484, which corresponded to cyanidin-3-O-galactoside (cyanidin-3-O-glucoside) and hydrochloride derivatives of these anthocyanins; fragmentation of the mass spectrum for the main peak of fractions 20–22 and 26–27 gave two main fragments: 417 and 453, which similarly corresponded to cyanidin-3-O-arabinoside (cyanidin-3-O-xyloside) and hydrochloride derivatives. The chromatograms of each analyzed sample and their peak identification and included in the Supplementary Materials (Figure S3). Due to the fact that cyanidin-3-O-galactoside and cyanidin-3-O-glucoside have the same molecular weight, as well as a pair of anthocyanins (cyanidin-3-O-arabinoside and cyanidin-3-O-xyloside), the HPLC method was used to confirm the composition.

Such individual anthocyanin extraction and purification procedures can further reveal and correlate the mechanism of extracts’ antiradical action to their chemical composition and individual components.

### 2.2. Chemiluminescent Activity of Investigated Compounds

**Figure 1:**
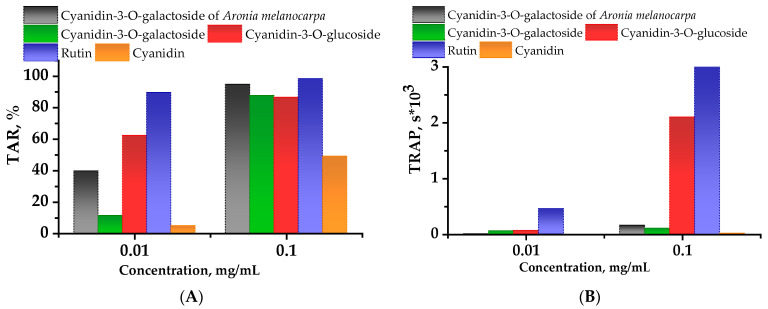
Chemiluminescent intensity attenuation (**A**) (TAR—total antioxidant reactivity) and time (**B**) (TRAP—total reactive antioxidant potential) vs. the concentration of the studied compounds. Values obtained from the quenching of luminol-enhanced chemiluminescence (CL).

Analysis of AAPH (2.2′-azobis(2-amidinopropane) dihydrochloride) chemiluminescent activity of the studied substances revealed a pronounced ability to reduce the chemiluminescence (CL) intensity. At concentrations of 0.1 mg/mL and higher, the difference in the total antioxidative capacity of the tested substances was not significant, and along with the rutin, the strong antioxidant of flavonoid origin, the investigated compounds showed high values of radical scavenging activity. At 0.01 mg/mL concentrations, the Aronia anthocyanin extract and the cyanidin-3-O-glucoside (Cy-3-Glu) solution in regard to CL attenuation showed respectively 28.5% and 51.0% higher values than Cy-3-Gal solution (Figure 1A). At concentrations of 0.1 mg/mL, the latent period for Cy-3-Glu solution is 32 and 33 min longer than that for Cy-3-Gal Aronia and Cy-3-Gal in similar concentrations (Figure 1B). Based on CL kinetics, cyanidin showed a relatively lower ability to scavenge free radicals, probably because anthocyanidins are less stable than their glycosylated forms. A comparison of the CL curves obtained for different concentrations of the five tested substances shows that the total antioxidant reactivity (TAR) of the samples decreased in a series: rutin, Cy-3-Gal, Cy-3-Gal Aronia, Cy-3-Glu (the total reactive antioxidant potential (TRAP) of Cy-3-Glu is better than that of the Cy-3-Gal), cyanidin. At the same time, the total antioxidant capacity of the Cy-3-Gal Aronia extract in concentrations above 0.01 mg/mL achieved its maximum and was probably related to the contribution of the total amount of antioxidants in the extract. The analysis of the latent period revealed that individual anthocyanins had a longer interaction with radicals than Aronia-extract cyanidin-3-O-galactoside and cyanidin.

### 2.3. Extracts’ Effect on DPPH-Scavenging Activity

In this study, the activity of the cyanidin-3-O-galactoside extract of the *Aronia melanocarpa* berries, as well of the purified anthocyanins, was determined as the ability to capture DPPH (2.2-diphenyl-1-picrylhydrazyl) radicals (Table 5). The activity of Aronia Cy-3-Gal was compared to an antioxidant of flavonoid-originated rutin. A more active agent of radicals’ suppression usually resulted in a lower effective concentration of EC_50_.

The anthocyanins varied slightly in their ability to scavenge DPPH radicals. As shown in Table 5, DPPH scavenging activity was up 30.1% and 8.86% higher for Aronia Cy-3-Gal than the corresponding values of Cy-3-Gal and Cy-3-Glu standards.

### 2.4. In Vitro Tests Analyzing the Extracts’ Effect on Hemolysis of Rats

**Figure 2:**
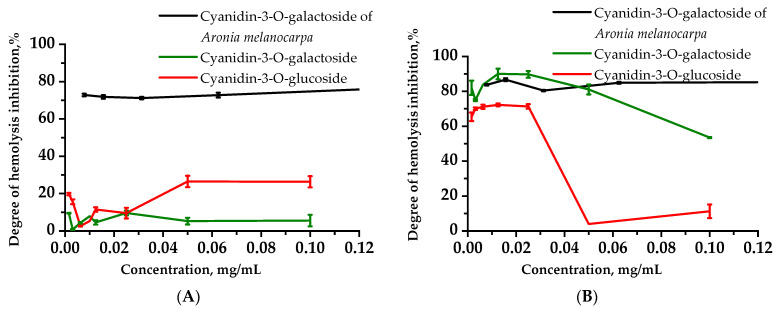
Osmotic (**A**) and peroxide (**B**) red blood cell hemolysis intensity vs. the concentration of cyanidin-3-O-galactoside Aronia extract, cyanidin-3-O-galactoside and cyanidin-3-O-glucoside using the model system of osmotic red blood cell damage and oxidative damage caused by Fenton reagent. Values are expressed as percent inhibition (mean ± S.D.).

Figure 2 presents the study results showing the ability of the tested compounds to inhibit osmotic and peroxide hemolysis, expressed as a percentage of the 100% control sample (without adding extracts). The results of the osmotic hemolysis analysis showed that cytoprotective anthocyanin action was observed within the studied concentration range; namely, it was less than 10% for Cy-3-Gal and less than 20% for Cy-3-Gal at the studied concentrations. The effects of Aronia Cy-3-Gal on red blood cells led to an increase in erythrocytic membrane resistance, with this effect increasing against the concentration growth. An in vitro evaluation of the effects of the studied compounds on erythrocyte membranes found that Aronia Cy-3-Gal at a concentration of 0.05 mg/mL exhibited higher ability (45.5% and 66.7%, respectively) to stabilize rat erythrocyte membranes for osmotic hemolysis than individual Cy-3-Glu and Cy-3-Gal anthocyanins (Figure 2A).

When the Cy-3-Glu and Cy-3-Gal specimen concentrations were more than 0.025 mg/mL, the red blood cell (RBC) suspension showed lower protection capacity against peroxide hemolysis. The effects of Cy-3-Glu on RBC membranes were 90% and 70% lower than those of Aronia Cy-3-Gal and Cy-3-Gal, respectively (Figure 2B). When the concentration of anthocyanin-containing Aronia fraction was 0.1 mg/mL, it was 73.7% and 31.5% more active against peroxide hemolysis than Cy-3-Glu and Cy-3-Gal, respectively.

Thus, the studied compounds showed their best membrane-stabilizing activity against oxidative degradation of RBCs by the Fenton reagent at concentrations of up to 0.025 mg/mL, where hemolysis inhibition levels of up to 72%, 90% and 86% were observed for Cy-3-Glu, Cy-3-Gal and Aronia Cy-3-Gal, respectively. The cyanidin-3-O-galactoside extract of *Aronia melanocarpa* exhibited the highest protection against osmotic damage to blood cells.

### 2.5. Cytoprotective Activity via In Vitro Experiments

**Figure 3:**
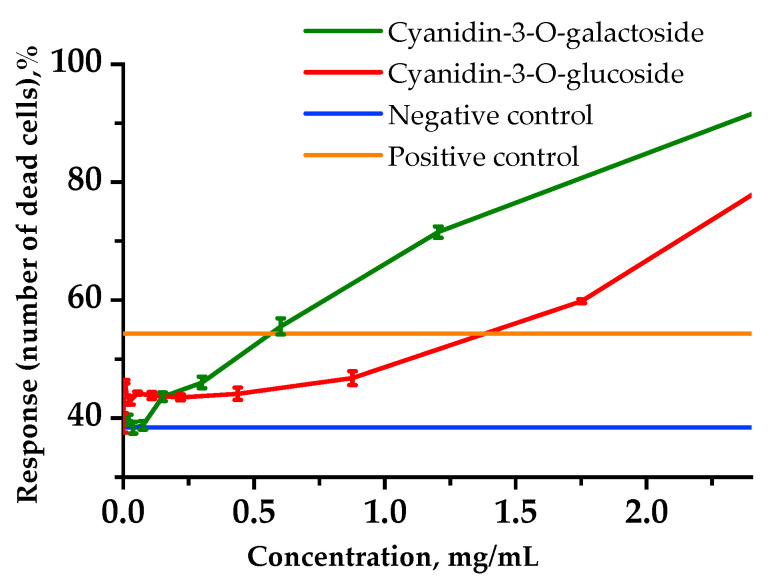
Cytoprotective activity of anthocyanins regarding the lymphoblastic RPMI-1788 cell line.

The cytoprotective activity of anthocyanins was studied in vitro using a human lymphoblast RPMI-1788 cell line (Figure 3). Cyclophosphamide (CP) as a cytostatic drug inhibited cell vitality. For example, in a positive control medium with CP at 1.25 mg/mL, the number of dead cells increased to 54.3%.

As for the human B lymphocyte cell line affected by CP, this revealed the cytoprotective activity within the range of up to 992 and 649 µg/mL for Cy-3-Glu and Cy-3-Gal, respectively.

### 2.6. Hematology Analysis of Rats’ Peripheral Blood

**Figure 4:**
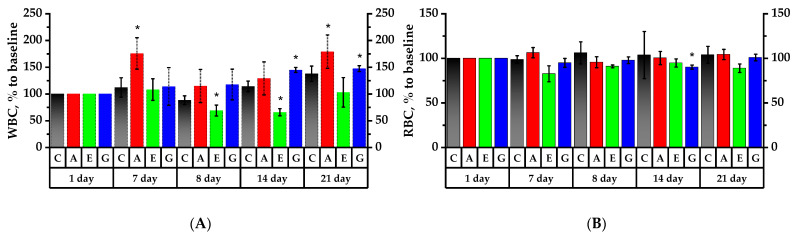

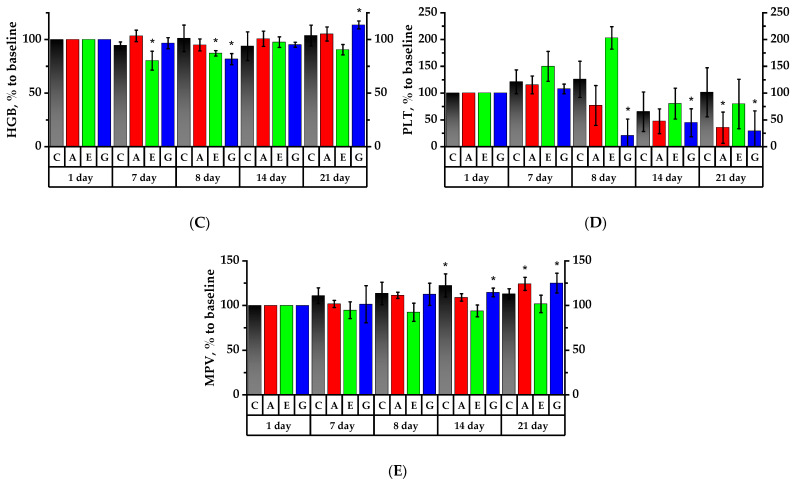
Main hematology results for whole blood of lab animals. The corresponding baselines for each group on the 1st day of the experiment are taken as 100%. (**A**) White blood cell (WBC) count; (**B**) red blood cell (RBC) count; (**C**) hemoglobin (HGB) concentration; (**D**) platelet (PLT) count; (**E**) mean platelet volume (MPV). * *p* < 0.05, significance of differences in animal groups compared to the 1st day of the experiment.

The prophylactic administration of Cy-3-Gal Aronia extract increased the total number of white blood cells (WBCs) from 6.58 ± 1.00 to 11.56 ± 1.13 × 10^3^ c/µL, which was reliably higher than the values obtained for the Control Group (8.45 ± 1.02 × 10^3^ c/µL) and Group G (9.06 ± 3.03 × 10^3^ c/µL). In Group G, WBC count in response to CP had an increase in quantity of 17.5% by the 8th day and 44.7% by the 14th day (*p* < 0.05). On the contrary, decreases of 34.8% in Group A and 36.3% in Group E (*p* < 0.05) were observed on the 8th day of the experiment and continued for 7 days in response to the CP (Figure 4A). On the 21st day, WBC count in Group E tended to increase. WBC count in Group A reliably increased to quantities of the 7th day, proving the recovery of leukograms in this group (*p* < 0.05).

**Figure 5:**
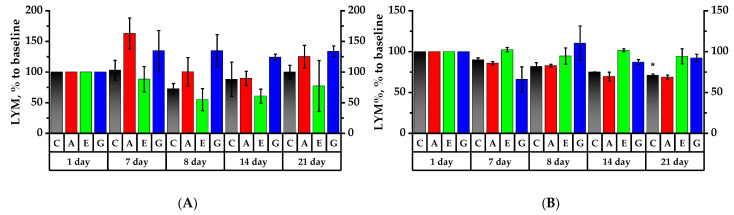

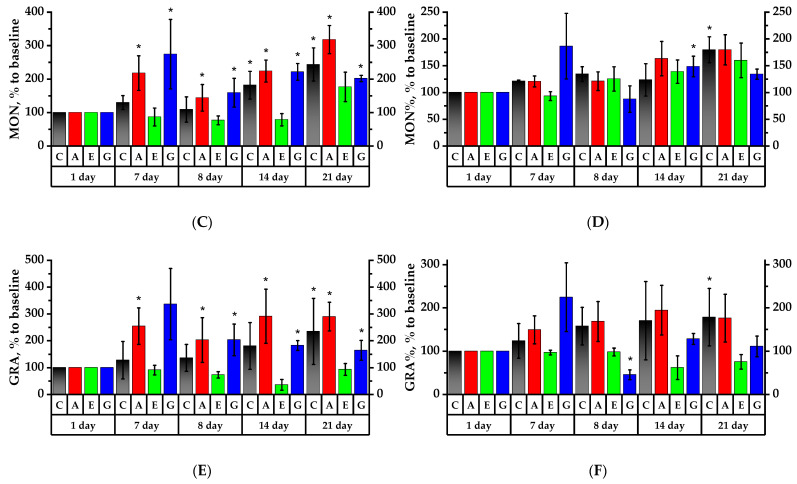
Leukocyte profile. The corresponding initial values for each group in the 1st day of the experiment are accepted as 100%. (**A**) Lymphocyte (LYM) count; (**B**) ratio of lymphocytes to total leukocytes (LYM%); (**C**) monocyte (MON) count; (**D**) ratio of monocytes to total leukocytes (MON%); (**E**) neutrophil granulocyte (GRA) count; (**F**) ratio of granulocytes to total leukocytes (GRA%). * *p* < 0.05, significance of differences in animal groups compared to the 1st day of the experiment.

Lymphocyte analysis (LYM) also revealed an increase in the number of cells in the group of rats receiving Aronia anthocyanins proactively. Similar to leukocytes, there was a downward trend in the number of lymphocytes in the Control Group, in Group E and in Group A after 8 and 14 days of the experiment (Figure 5A). On the 21st day, LYM count increased by 25.3% over 1 day in Group A, by 33.7% in Group G, and by 22.1% in Group E over 8 days of the experiment (*p* < 0.05). On the 14th and 21st days of the experiment, LYM count in Group G was reliably higher than that in Group A by 3.5% and 8.3%, respectively (*p* < 0.05), meaning that cyanidin glycosides stimulated the functional metabolic activity of rat lymphocytes.

The monocyte test (MON) found an increase in monocytes of 2.2 times after the anthocyanin fraction of *Aronia melanocarpa* was received by rats (*p* < 0.05) for 7 days and 2.8 times after rats received the anthocyanin solution (*p* < 0.05) for 7 days. The monocyte count increased reliably during the test in Group A, and by the 21st day, it was 2.2 times that on the 8th day of the test.

In the Control Group, a 1.2 times decrease in the monocyte count was observed on the 8th day; however, it recovered within 7 days. The study of the monocyte dynamics did not reveal a significant effect of Echinacea tincture on rat blood monocytes. By the 21st day of the experiment, there was a reliable 2.4 times increase in the monocyte count in the Control Group, as well as an increase in Group G by 2 times (Figure 5C).

The hematological test also revealed a significant increase in the granulocyte count (GRA) in the group of rats fed with *Aronia melanocarpa* extract and anthocyanin solution for 7 days by 2.8 and 2.1 times, respectively (*p* < 0.05). GRA count in these groups decreased in response to CP introduction, but it was definitely higher than the value in the Control Group (*p* < 0.05). On the 14th day of the experiment, GRA count in Group G was definitely 1.5 times higher than that in Group E. On the 21st day, GRA count increased in the Control Group, as well as in Groups A and G (*p* < 0.05). In Group E, the value was decreased by 2.8 times in 7 days after cytostatic administration; on the 21st day of the experiment, GRA count recovered—the value was only 1.1 times lower compared to the 1st day (Figure 5E).

The relative lymphocyte count test (LYM%) showed that in the Control Group the value tended to decrease and definitely decreased by 29% by the 21st day of the experiment (Figure 5B). In Group A, similar to the Control Group, the value showed a decreasing tendency on the 7th day by 14.2%, on the 8th day of the experiment by 17.2%, on the 14th day by 30.4% and on 21st day by 31.2% compared to the 1st day of the experiment. The relative contents of lymphocytes in Group E and Group G on the 14th day of the experiment were definitely higher compared to that in Group A by 32% and 12.8%, respectively. In Group G, the relative content of lymphocytes was reliably higher on the 14th day of the experiment by 17.6% and on day 21 by 23.5% compared to Group A (*p* < 0.05).

The relative monocyte count test (MON%) showed a significant increase in the value by 79.8% under control by the 21st day compared to the 1st day of the experiment. The MON% value did not change in response to the CP injection in Group A and increased by 1.6 times after the 14th day of the experiment compared to the 1st day. In Group E, there was a significant 32.8% increase in the value by the 14th day. On the 21st day, the value increased by 66.7% compared to that on the 7th day of the experiment; there was also a tendency for the value to increase by 60% by the 21st day of the experiment. In Group G, MON% was significantly higher by the 14th day of the experiment by 48.6% compared to the 1st day and by 61% compared to the 8th day of the experiment. On the 21st day, the MON% value significantly increased by 46.6% compared to the 8th day; however, it remained below the MON% value in Group A by 45.5% (*p* < 0.05) (Figure 5D).

The relative neutrophil count test (GRA%) revealed that animals fed with Cy-3-Gal Aronia fraction and individual anthocyanins for 7 days showed 1.5 and 2.2 times increases in the relative neutrophil count, respectively. In Group G, the value significantly decreased by 54.3% on the 8th day compared to the 1st day (*p* < 0.05) and increased by 82.3% on the 14th day as compared to the 8th day. It increased by 65.3% on the 21st day (*p* < 0.05).

In Group A and in the Control Group, the relative neutrophil count increased on the 8th day. In the Control Group, on the 21st day, there was a significant 2.2 times increase in the GRA% value compared to the 1st day. In Group A, GRA% reached a 1.7 times higher value compared to the 1st day. On the contrary, in Group E, the GRA% value decreased and reached its minimum by the 14th day of the experiment (1.6 times lower compared with the 1st day) (Figure 5F).

In the group of rats fed with Cy-3-Gal Aronia for 7 days, the RBC count was observed to increase by 6.3%, while this value decreased by 17.3% in Group E and by 5.1% in Group G (Figure 4B). Seven days after cytostatic injection, a 13.6% RBC decrease was observed in Group G as compared to the values of the 1st day (*p* < 0.05). On the 21st day of the experiment, the value significantly increased by 10.8% as compared to the 14th day (*p* < 0.05). The changes in RBCs following the Cy-3-Gal Aronia effect were not so significant; namely, 24 h after the CP injection, a decreasing trend in value was observed, but in 7 days it recovered and even tended to increase by the 21st day of the experiment.

In response to cytostatic injection, the hemoglobin (HGB) concentration decreased by 1.2 times in Group G (*p* < 0.05); however, it significantly increased after 7 days. By the 21st day of the experiment, the concentration of hemoglobin in the rats’ blood of Group G significantly increased by 13.7% compared to the 1st day. Injection of Cy-3-Gal Aronia into rats resulted in an increase in hemoglobin concentration in the blood after 7-day prophylactic administration. After a decrease in response to the CP effect, the hemoglobin concentration in this group was completely restored after a week (Figure 4C).

The platelet (PLT) count test in the Control Group revealed a PLT count decrease on the 7th day after the CP injection and recovery to initial values by the 21st day (Figure 4D). In Group E, there was a tendency for the PLT count to increase by 1.5 times on the 7th day and by 2 times on the 8th day compared to the 1st day. However, a week after the CP injection, PLT count decreased by 2.5 times (*p* < 0.05). In Group A and Group G, there was a PLT decrease on the 8th, 14th and 21st days in comparison with the 1st day of the experiment (*p* < 0.05)

In the Control Group the mean platelet volume (MPV) Test identified a significant 22.4% increase on the 14th day of the experiment compared to the 1st day. In Group A, mean platelet volume significantly increased by 24.2% on the 21st day compared to the 1st day, by 22.5% compared to the 7th day of the experiment and by 12.8% compared to the 8th day (*p* < 0.05). In Group E, one can note a tendency to decrease by 5.3% on the 7th day and by 7.6% on the 8th day in comparison with the 1st day of the experiment; on the 21st day, almost a 2% increase in MPV can be noted. In Group G, the value on the 8th day of the experiment was significantly 1.1% higher compared to Group A and 20.1% higher compared to Group E (*p* < 0.05). On the 14th day of the experiment, the MPV value significantly increased in the Control Group and in Group G in comparison with the 1st day by 22.4% and 14.6%, respectively. On the 21st day, MPV increased by 24.2% and 25.1% compared to the 1st day in Groups A and G, respectively (*p* < 0.05) (Figure 4E).

### 2.7. Leukocyte Phagocytic Activity Test

**Figure 6:**
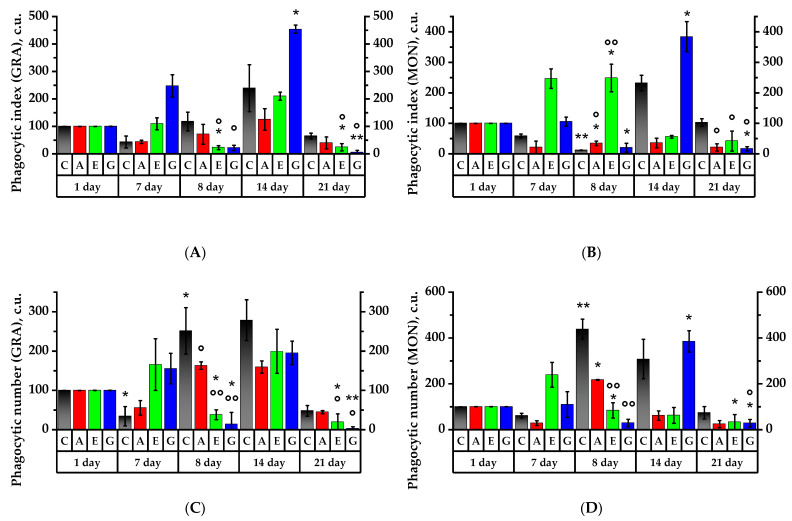

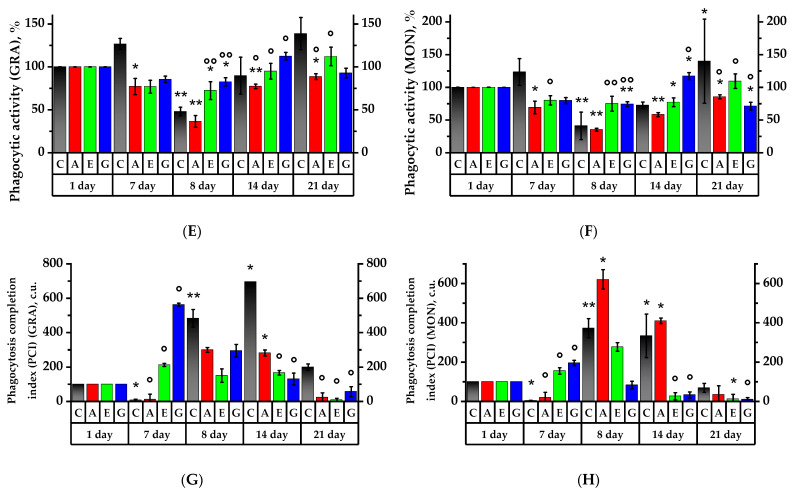
Phagocytic activity of neutrophil granulocytes and rat peripheral blood monocytes. Values (phagocytic index (**A**,**B**), phagocyte number (**C**,**D**)) are expressed in conventional units (c.u.); phagocytic activity (**E**,**F**) is expressed in %; phagocytosis completion index (**G**,**H**)) is expressed in c.u. * *p* < 0.05, ** *p* < 0.01, significance of differences compared to the 1st day of the experiment; ° *p* < 0.05, °° *p* < 0.01, significance of differences between the animal groups as compared to the Control Group.

The analysis of the phagocytic activity values offers the opportunity to identify the influence of plant substances and their components on the functional activity of cells of the rat immune system. The phagocytic index test (PI) of neutrophil granulocytes of the rat peripheral blood showed that the Control Group had a tendency for this value to decrease by up to 57% as compared to the 1st day of the experiment (Figure 6A). There was an 18% increase on 8th day and a 70.5% increase on the 21st day.

In Group A, neutrophil PI declined by 56% in response to the 7-day dosing and increased by 23.5% 24 h after CP was injected. By the 14th day of the experiment, the PI had increased by 25.1% compared to the 1st day of the experiment. In Group E, there was a slight increase in PI on the 7th day but significant decrease by 6.9 times (on the 8th day) compared to the Control Group (*p* < 0.05), indicating a decrease in digestive capacity of neutrophil granulocytes. In Group G, a 1.5 times PI increase was found on the 7th day; 24 h after CP was injected, neutrophil PI in Group G was reliably reduced by 11.5 times as compared to the 7th day of the experiment (*p* < 0.01) and was reliably (11 times) lower than that in the Control Group (*p* < 0.05) and 1.6 times lower as compared to Group A (*p* < 0.01); on the 14th day of the experiment, in Group G, the PI was reliably 1.8 times higher in comparison to that on the 7th day (*p* < 0.05).

The results of PI of monocytes in rat peripheral blood showed a reliable decrease of 88% in response to the cytostatic dosing in the Control Group (*p* < 0.01) (Figure 6B). In 7 days, there was a reliable 19.7 times increase as compared to the 8th day of the experiment, and after 21 days, there was an 8 times increase (*p* < 0.05). In Group A, on the 8th day of the experiment, monocyte PI declined almost 3 times compared to the 1st day of the experiment (*p* < 0.05) but was 4.8 times higher than that in the Control Group (*p* < 0.05). In Group E, the monocyte PI was 2.5 times higher on the 8th day than on the 1st experimental day (*p* < 0.05) and was reliably (15.3 times) higher than that in the Control Group (*p* < 0.01) and 3.2 times higher as compared to Group A (*p* < 0.05). On the 14th day, the value in this group was reduced by 4.5 times as compared to the 8th day (*p* < 0.05). In Group G, the monocyte PI declined by 80% a day after cytostatic was injected compared to the 1st day of the experiment (*p* < 0.05), and on the 8th day of the experiment the monocyte PI was 2.7 times lower as compared to Group A (*p* < 0.05). By the 14th day, the PI value in Group G had increased by 19 times compared to 8th day of experiment (*p* < 0.01). By day 21, PI monocytes in the Group G reliably decreased 23 times compared to the 14th day (*p* < 0.01) (Figure 6B).

The study of the phagocytic number (PN) behavior pattern on the 8th experimental day revealed a reliable increase in the number of bacterial particles captured by one neutrophil and monocyte in the Control Group by 2.5 times and 4.5 times, respectively, as compared to the 1st day (*p* < 0.05) and then a 5.2 times reduction on the 21st day as compared to the 8th day (*p* < 0.05) (Figure 6C,D). In Group A, on the 8th day of the experiment, PN value reliably increased by 1.6 and 2.2 times for granulocytes and monocytes, respectively, as compared to the 1st day of the experiment (*p* < 0.05). By the 14th day of the experiment, the neutrophil granulocyte increase was 2.9 times greater than its previous value as of the 7th day (*p* < 0.05) (Figure 6C). Within a day of cytostatic injection in Group E, compared to Group A, the PN value became 5.4 times lower (*p* < 0.01) and significantly by 2.6 and 4.4 times lower as compared to the 1st and the 7th days of the experiment (*p* < 0.05). On the 14th day of the experiment, the PN value increased by 1.2 and 5.3 times compared to the 7th and 8th experimental days, respectively (*p* < 0.05). It is arguable that on the 7th day of *Echinacea purpurea* herb tincture intake, phagocyte activity of neutrophils increased, as the average number of bacteria ingested by a single neutrophil increased.

In Group G, 24 h after CP injection, the number of bacterial particles captured by granulocytes and monocytes dropped dramatically by 86.3% and 70%, respectively, as compared to the 1st day (*p* < 0.05) and was 14 and 12.4 times lower, respectively, than that in Group A (*p* < 0.01). On the 14th day in Group G, the PN increased reliably for monocytes and was reliably 6.3 times higher as compared to the Group A (*p* < 0.05) (Figure 6D). In granulocytes, the PN value tended to increase by 95% as compared to the 1st day of the experiment. These results show a strong effect of anthocyanins on phagocytes, especially in rat peripheral blood monocytes.

On the 8th experimental day, granulocytes and monocytes of the Control Group showed an increase in the number of bacterial particles by 2.5 (*p* < 0.05) and 4.4 times, respectively (*p* < 0.01). As compared to the Control Group, in experimental Groups A, E and G, the number of captured *E. coli* particles decreased under immunosuppressive conditions. Seven days after CP injection, the ability to ingest bacterial particles increased in the granulocytes of rat groups fed with Echinacea tincture and anthocyanin solution, and in the Control Group, it did not change. In the granulocytes of the rat group receiving the anthocyanin Aronia fraction, this ability did not change. A similar effect occurred for monocytes in Group G receiving the Cy-3-Gal and Cy-3-Glu mixture.

The study of phagocytic activity (PA) revealed a reliable decrease in the number of active neutrophils and monocytes in the Control Group in response to CP exposure—by 52% and 59%, respectively, as compared to the 1st day of the experiment (*p* < 0.01) (Figure 6E,F). Later the values were spontaneously restored, and by the 21st day, the PA values of granulocytes and monocytes reliably increased by 2.9 and 3.4 times, respectively, as compared to the 8th experimental day (*p* < 0.05).

Similar to the Control Group, the PA values of neutrophils and monocytes in Group A on the 8th day of the experiment were reliably reduced by 63.5% and 64.8%, respectively, compared to the 1st day (*p* < 0.01). On the 14th day of the experiment, the value increased by 40.1% in granulocytes and 23% in monocytes compared to the 8th day (*p* < 0.05). On the 21st day of the experiment in this group, the values increased reliably by 11.3% and 27.2%, respectively, as compared to the 14th day (*p* < 0.05). According to the results obtained, it can be argued that there was a decrease in the manifestation of immunosuppression under the influence of the anthocyanin fraction of black chokeberry extract.

In Group E, on the 8th day, neutrophil PA decreased by 28% (*p* < 0.05) compared to the 1st day of the experiment, while the number of active granulocytes and monocytes was significantly higher compared to the Control Group—by 72.5% and 98%, respectively (*p* < 0.01). In 7 days, neutrophil PA in Group E increased, while the Control values increased by 21% (Figure 6E). At the same time, the PA of monocytes did not change and became significantly lower by 22.9% as compared to the 1st day (*p* < 0.05). On the 21st day of the experiment, the PA of neutrophils and monocytes in Group E significantly increased compared to the 8th day of the experiment by 55% and by 32.15%, respectively (*p* < 0.05), which was proof of the fact that the use of *Echinacea purpurea* tincture contributed to the restoration of phagocytic activity.

On the 8th day of the experiment in Group G, the PA value of granulocytes and monocytes was significantly lower compared to the 1st experimental day—by 17.5% (*p* < 0.05) and 25.8% (*p* < 0.01), respectively. However, in Group G, granulocyte PA was significantly higher (2.3 and 2.2 times, respectively) compared to the Control Group and Group A (*p* < 0.01), and also significantly higher by 10.2% compared to Group E (*p* < 0.05), indicating a rapid immune response to CP effects. On the 14th day in Group G, the PA of neutrophils and monocytes significantly increased by 30% (*p* < 0.05) and by 43% (*p* < 0.01), respectively, as compared to the 8th day. Moreover, neutrophil PA was significantly higher compared to Group A and Group E by 35.1% (*p* < 0.01) and 17.5% (*p* < 0.01), respectively, and monocyte PA was 40% higher as compared to Group E (*p* < 0.01) (Figure 6F).

The study of the phagocytosis completion index (PCI) in the Control Group showed a 13 times decrease for granulocytes and 26 times decrease for monocytes (*p* < 0.05) by the 7th day of the experiment as compared to the 1st day, while in Groups E and G, the PCI increased by 2.1 and 5.6 times, respectively (Figure 6G,H). It is significant that by the 7th day, the PCI values for all experimental groups were significantly higher than the control values (*p* < 0.05)—by 3, 50 and 71 times for Groups A, E and G, respectively. On the 8th day of the experiment in the Control Group, the PCI of granulocytes and monocytes increased by 4.8 and 3.7 times, respectively (*p* < 0.01), as compared to the 1st day. On the 14th day, the ingestion of bacteria by granulocytes was reduced to a minimum, and PCI in the Control Group increased by 1.4 times (*p* < 0.05). By the 21st day, in the Control Group, PCI for granulocytes was higher than that of the experimental groups (*p* < 0.05) (Figure 6G).

In Group A, the PCI of granulocytes increased by 26 times on the 8th day of the experiment as compared to the 7th day of the experiment (*p* < 0.05), and the PCI of monocytes increased by 6.2 and 32.2 times as compared to the 1st and 7th days of the experiment (*p* < 0.05); at the same time, increases in microbicidal properties were observed after two hours of incubation with microorganisms. On the 14th day, PCI of granulocytes was 2.8 times higher and 24 times higher (*p* < 0.05) than that on the 1st and 7th days of the experiment. The monocyte PCI was 4.1 times higher and 21.2 times higher as compared to the 1st and 7th days of the experiment, respectively (*p* < 0.05). It is definite that Group A monocytes under immunosuppressive conditions have a reactive response, and microbicidal activity to *E. coli* is enhanced.

In Group E, the PCI of monocytes tended to increase by 8th day (Figure 6H). The PCI values for granulocytes and monocytes on the 14th day of the experiment were reliably below the values of the Control Group by 2.3 and 4.3 times, respectively (*p* < 0.05). However, in Group E, on the 21st day of the experiment, the PCI of granulocytes was reliably reduced by 8.5 times as compared to the 1st day of the experiment (*p* < 0.05).

In Group G, on the 8th day, the PCI of monocytes was reliably (7.1 times) lower than that in Group A (*p* < 0.05). By the 14th day, the PCI of granulocytes in Group G was lower than that in the Control Group and Group A by 5.4 and 4.8 times, respectively, and the PCI of monocytes was lower than that in the Control Group and Group A by 5.8 and 11.9 times, respectively (*p* < 0.05). The PCI of granulocytes was 3.9 times higher than the value in Group E by the 21st day, and PCI of monocytes was 3.4 times lower than the value in Group A (*p* < 0.05).

### 2.8. Study of Spontaneous and Activated Neutrophil Chemiluminescent Activity

**Figure 7:**
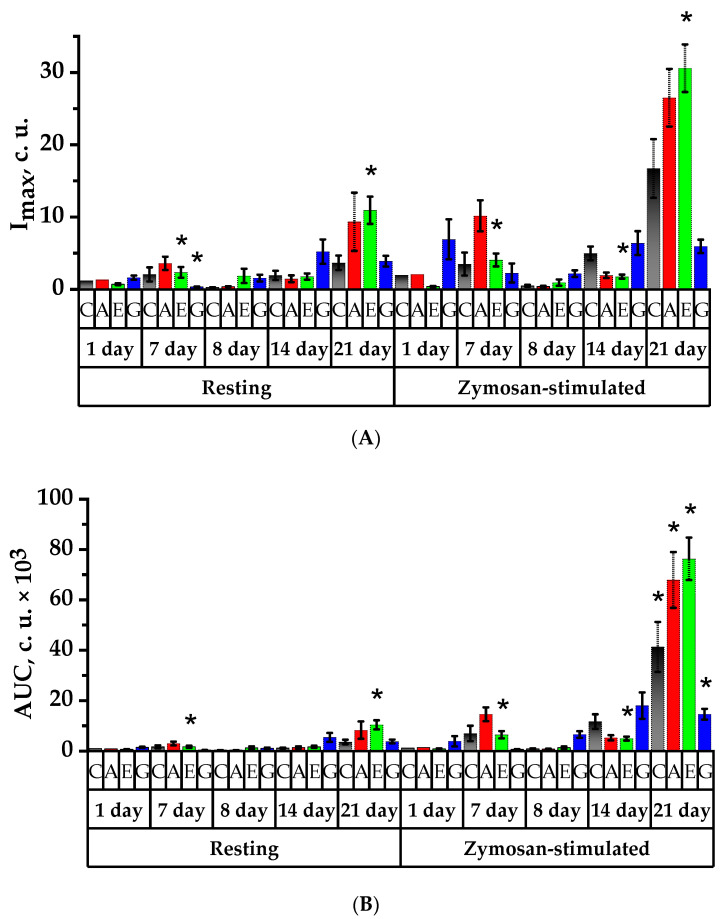

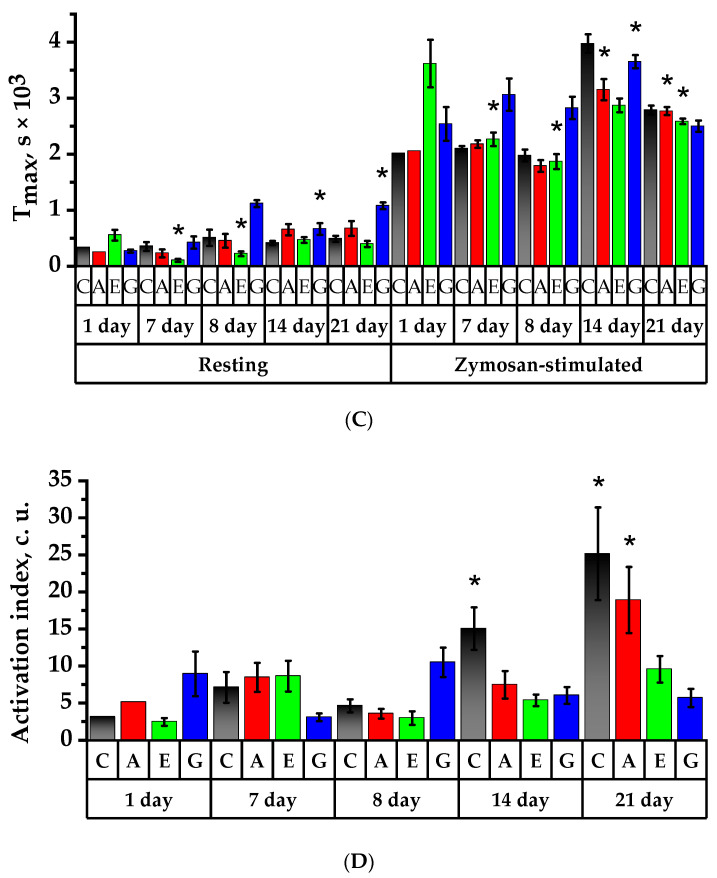
Spontaneous and zymosan-activated CL of rat neutrophils: (**A**)—CL intensity (I_max_, c.u.); (**B**)—area under the CL curve (AUC, c.u.); (**C**)—the time showing peak CL (T_max_, c.u.); (**D**)—activation index (I_act_, c.u.). * *p* < 0.05, significance of differences in animal groups compared to the 1st day of the experiment.

The intrinsic (spontaneous) neutrophil chemiluminescent activity study showed an increase in maximum “metabolic burst” (I_max_) by 2.8 and 3.7 times on the 7th day when the rats were administered Aronia anthocyanin fraction and Echinacea tincture, respectively (Figure 7A). In Groups A and E, the formation of reactive oxygen species (ROS) in the presence of zymosan increased by 5.1 and 13.5 times (*p* < 0.05), respectively. In Group G the maximum “metabolic burst” increased by 2.5 times in the presence of zymosan for 24 h, and on the 7th day it increased by 8.5 times; however, as compared to the 1st day, the intensity of the intrinsic ROS formation in Group G decreased on the 7th day (*p* < 0.05).

Twenty-four hours after immunosuppression was simulated in Group G, a 5.8 times increase in the maximum of the “metabolic burst” of neutrophils was observed as compared to the values before the CP was injected (*p* < 0.05). In Group A, a decrease in the total number of ROS relative to the level of 7th day of spontaneous and induced CL was observed by 12 and 33.7 times, respectively (*p* < 0.05). In the Control Group and Group E, there was a decrease in the intensity of ROS production in response to the introduction of zymosan by 8.5 and 4.7 times, respectively.

On the 14th day of the experiment, an increase in the intrinsic intensity of neutrophils’ CL was observed, in the Control Group by 10.3 times, in Group A by 4.7 times and in Group G by 3.5 times. The intensity of the zymosan-induced luminescence in the same groups increased by 12.3, 6.2 and 3 times, respectively. No significant changes in these parameters were observed in Group E (Figure 7A).

The maximum of the “metabolic burst” was observed on the 21st experimental day in Groups A and E; at the same time, an increase was noted by 6.8 and 6.5 times in spontaneous luminescence intensity and by 14.3 and 18.4 times in zymosan-induced luminescence intensity, respectively (*p* < 0.05). The parameters of zymosan-induced ROS production by rat neutrophils increased by 4.7 times on the 21st day for the Control Group and by 2.9, 2.8 and 1.5 times for Groups A, E and G, respectively.

Area under the chemiluminescence curve (AUC) analysis (Figure 7B) showed that during the spontaneous and induced CL reaction, there was an increase in the level of ROS production by neutrophils in Group E. After animals in Group A were injected with CP, the total number of ROS for background and zymosan-induced CL reactions decreased by 16 and 25 times, respectively, and there was a 1.4 times decrease for the induced CL reaction in Group E. In Group G, on the contrary, the area under the CL curve increased by 14.6 times (*p* < 0.05).

On the 7th day after CP-induced immunosuppression, an increase in the AUC was observed as compared to the 8th day in the Control Group by 7.5 times in the case of spontaneous and 17.3 times in the case of zymosan-induced CL; the AUC values of spontaneous CL in Groups A, E and G increased by 7, 1.4 and 5.8 times, respectively. The total number of ROS increased on the 21st day in all the studied groups relative to the day of cytostatic injection, and it significantly increased as compared to the 14th day in Group A by 6.7 and 13.3 times and in Group E by 6.9 and 15.9 times in the case of background and zymosan-induced CL, respectively (*p* < 0.05).

The analysis of the T_max_ value (Figure 7C) in Group E in the period before CP injection showed an acceleration of the curve of spontaneous and induced peripheral blood neutrophil CL, less than in Group G (*p* < 0.05). In the period after CP injection, there was a slowdown in the T_max_ value of spontaneous CL of neutrophils by 2.2 times in Group E and by 2.7 times in Group G, and, on the contrary, there was a 1.2 times reduction in the period of induced neutrophil CL in Group A (*p* < 0.05).

On the 14th day of the experiment, the time to reach the maximum neutrophil CL intensity at the resting state in Group G was reduced by 1.7 times, whereas when zymosan was promoted, the time to reach the maximum neutrophil CL intensity increased in all the groups under study—by 2.0, 1.8, 1.5 and 1.3 times in Control, A, E and G Groups, respectively. On the 21st day of the experiment, the time of reaching the maximum CL of spontaneous CL of neutrophils in Group G was reduced by 1.6 times, and, at the same time, the induced CL curve growth was accelerated significantly in the Control Group and in Group G by 1.4 and 1.5 times, respectively (*p* < 0.05).

In the period after CP was injected, there was a tendency for the value of neutrophil I_act_ to decrease (Figure 7D); however, it was observed to be increased by 3.4 times in Group G. On the 14th day of the experiment, the activation index increased in the Control Group by 3.2 times, in Group A by 2.1 times and in Group E by 1.8 times and decreased in Group G by 1.7 times. By the 21st day of the experiment in Group A, the I_act_ value reached its maximum and was significantly higher than that on day 8 by 5.3 times (*p* < 0.05). In Group E, the I_act_ value increased 1.8 times relative to day 14 and was also significantly 3.2 times higher as compared with the 8th day (*p* < 0.05). In Group G, on the contrary, the I_act_ value decreased 1.8 and 1.1 times as compared to the 8th and 14th days, respectively (*p* < 0.05).

In conclusion, in the rats of Group A, similarly to Group E, in the period before the CP injection, a change in the CL response kinetics of neutrophil peripheral blood was observed when stimulated with zymosan to increase the light sum and amplitude of the CL curve.

In the period after CP administration, the level of background and zymosan-stimulated CL was observed to decrease. It should be noted that in Group G, the ability of blood neutrophils to increase the production of secondary ROS in response to zymosan stimulation in vitro remained unchanged, as evidenced by the I_act_.

In 7 days, changes in the kinetics of the zymosan-induced CL response of blood neutrophils remained in the control rats and rats of Group G, and high levels of primary and secondary ROS generation were determined; high values were observed mainly for group G at rest. It is worth noting that control animals had an increased ability of blood neutrophils to enhance secondary ROS generation in response to zymosan stimulation in vitro, as evidenced by their cell activation index. At the same time, the increase in secondary ROS generation could lead to the peroxidation of cell membrane lipids and affect the cell functional status.

On day 21, neutrophils stimulated the generation level of both superoxide radicals and secondary ROS in Groups A and E. The kinetics of CL response in Group G maintained the values obtained on the 14th day. The relative values of primary and secondary ROS generation by the blood neutrophils of the Control Group in vitro during zymosan stimulation showed an increase in secondary ROS generation.

### 2.9. Lipid Peroxidation Study

**Figure 8:**
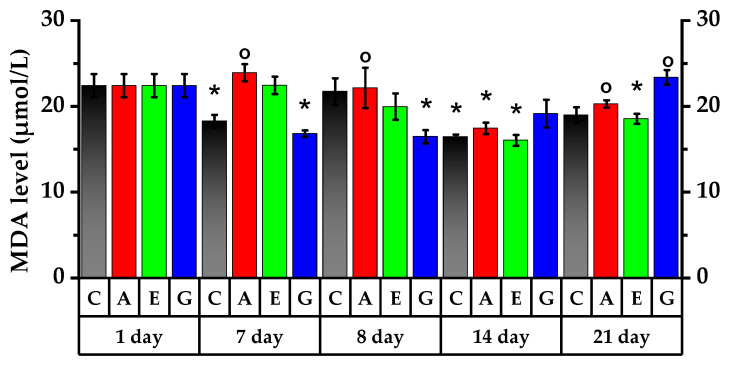
Malondialdehyde (MDA) level of peripheral blood of rats. * *p* < 0.05, significance of differences in animal groups compared to the 1st day of experiment. ° *p* < 0.05, significance of differences between animal groups compared to Control Group.

Rat lipid peroxidation (LPO) was investigated to determine the degree of substances’ impact on the antioxidant system of the body (Figure 8). On the 7th day of the experiment, RBCs in experimental rats showed a decrease in malondialdehyde (MDA) level, which was confirmed in the Control Group and Group G.

After CP single dosing, the LPO product was accumulated in the Control Group (increased by 19% compared to the 7th day of the experiment), confirming LPO process enhancement (*p* <0.05). Elevated MDA is representative of reduced antioxidants in control rats that effectively detoxify LPO products. MDA in Group G red blood cells was reliably 26.6% lower as compared to the Control Group (*p* < 0.05).

Gradually, after 7 days, rats receiving Aronia anthocyanin faction administration, rats receiving Echinacea tincture administration and rats in the Control Group exhibited a reliable decrease in MDA by 27%, 24.5% and 32%, respectively (*p* < 0.05). LPO decrease was observed on the 21st day in Groups A, E and G (*p* < 0.05). On the 21st day, MDA content was reliably higher in Group G as compared to the other three groups under study. On the 21st day, MDA concentration in Groups A and G was reliably higher by 9.3% and 26.1% than that in Group E; however, MDA concentration in Group A was reliably less than that on the 8th day of the experiment.

This study showed that the accumulation of the lipid peroxidation product MDA in the red blood cells of rats increased under immunosuppressive conditions, while administration of anthocyanins and Echinacea tincture prevented this process.

## 3. Discussion

In this study, ethanol extract and high-anthocyanin extract from *Aronia melanocarpa* berries were obtained. The component composition for each of the extracts was determined by HPLC and spectrophotometry methods. Anthocyanins in the resulting anthocyanin fraction increased by 9.8 times as compared to ethanol extract, with the major compound being cyanidin-3-O-galactoside, the content of which was 82.2%. By the method of column chromatography, we isolated a fraction of cyanidin-3-O-galactoside (Cy-3-Gal Aronia) with a purity of more than 93.7%, which was used in this study. Analysis of the free radical-scavenging activity of the extract by chemiluminescence found that the compounds of cyanidin-3-O-glucoside, as well as cyanidin-3-O-galactoside, had the greatest ability to inhibit the formation of free radicals, in agreement with the authors of [20].

A DPPH assay review showed that tested substances were able to react with DPPH radicals in the following descending order: rutin, Cy-3-Gal Aronia, cyanidin-3-O-glucoside standard and cyanidin-3-O-galactoside standard. The higher antioxidant activity of Cy-3-Gal Aronia in comparison with a similar standard can be conditioned by the presence of other antioxidants as impurities, for example, the phenolic fraction, which, as reported in [21,22], plays an essential role in neutralizing free radicals, thereby increasing its antioxidant capacity, as well their possible synergy, since, as reported in [23], the therapeutic effect of herbal medicines is often based on the synergistic effect of their mass components, and the portion of components in herbal medicines can influence the effect manifestation. In addition, the differences in the activity of anthocyanidins and anthocyanins in the ability to inactivate free radicals obtained in the DPPH test may be associated with the number and position of hydroxyl and methoxy groups, as well as sugar substitute availability. The results showed a correlation between the number of -OH groups in the B ring and the ability to scavenge free radicals of both anthocyanidins and anthocyanins [24].

Following the test of anthocyanin effects on rat red blood cell membranes in vitro allowed us to compare Cy-3-Gal Aronia to compounds capable of protecting the model membrane from oxidation more effectively than Cy-3-Gal and Cy-3-Glu. In [25], there is a description of the protective effect against oxidative damage occurring when anthocyanins are incorporated into the membrane and cytosol of cells.

The concentration range of Cy-3-Gal and Cy-3-Glu (up to 649 and 992 µg/mL) was revealed, whereby anthocyanins’ protective effect on the cell culture of RPMI-1788 lymphoblasts against CP was observed, and on the contrary, when concentration was increased, cell viability was noticed to be decreased. It seems probable that such a cytotoxic effect at high concentrations of cyanidins is due to the fact that, as illustrated by the authors of [26], anthocyanin metabolites can reduce cell viability basically by inhibiting cell proliferation.

In fact, the injection of cytostatic drugs, including CP, can inhibit the proliferation and differentiation of any cells that divide rapidly, such as immunocompetent cells [27,28].

According to the literature, after the herbal drugs’ introduction, the repair process restoration was accelerated [29,30]; polyphenol compounds are possible to proceed similarly. As illustrated in [31], when using *Echinacea purpurea* tincture affected by a single cytostatic injection, the total leukocytes decreased by 1.6 times, and leukogram improvement was observed by the end of the experiment. The resulting effect may be related to the ability of Echinacea, preferentially chicoric acid and echinacin, to stimulate bone tissue, bone marrow and hematopoietic stem cells [32]. According to Murthy et al. [33], the treatment of rats with Echinacea slightly changed the level of platelets.

In this study, the immunostimulating effect Cy-3-Gal Aronia was expressed in positive dynamics of rats’ leukograms. Thus, the total leukocytes increased 1.8 times by the 21st day; the level of monocytes increased 3.2 times and the level of granulocytes increased 2.9 times. That is, there was a shift in leukogram towards more monocytes and neutrophils and lower levels of lymphocytes. The anthocyanin mixture solution also exhibited an immunostimulating effect, similar to the cyanidin-3-O-galactoside fraction of black chokeberry. Under its influence, the level of leukocytes increased by 1.5 times, the level of monocytes increased by 2 times and the level of granulocytes increased by 1.6 times. The shift of leukogram of the rats’ group with anthocyanin solution was similar to that of the group with Cy-3-Gal Aronia.

In general, evaluating the influence of the investigated substances on the hematological values, it can be stated that the extract of black chokeberry enriched by the anthocyanin fraction as well as the solution of the anthocyanin mixture showed a reliable activating effect on the proliferation of leukocytes, including monocytes, lymphocytes and neutrophils, but exhibited an antiplatelet effect, which is consistent with the results of [34], while the well-known immunomodulator *Echinacea purpurea* tincture had less of an effect on increasing the level of leukocytes, but resulted in an increase in platelets. Moreover, black chokeberry extract enriched by anthocyanin fraction and anthocyanin solution had a stimulating effect on red blood cell and hemoglobin levels.

The study of neutrophil and monocyte phagocytic activity as well as neutrophil chemiluminescent activity revealed an improvement in the functional status of immune cells involved in the phagocytosis, as affected by Cy-3-Gal Aronia and a mixture of Cy-3-Gal and Cy-3-Glu. At the same time, the efficacy of anthocyanins was far greater than that of the Echinacea tincture, a well-known immunomodulator.

It is a common fact that *Echinacea purpurea* tincture [35] enhances phagocytosis due to the presence of alkaloids, carbohydrates (Echinacea heteroxylan), tannins, phenolic compounds, saponins and glycosides. Some data from publications known for anthocyanins prove their ability to activate S. *aureus* phagocyte engulfment [31], which is entirely consistent with the results obtained in our study. Activation of phagocytes with an increase in their effector potential (ingestion and digestive capacity, ROS generation) led to inflammatory reaction development aimed at pathogen elimination [36], in other words, strengthening barrier functions of the body. In immunosuppressive conditions, phagocyte activity was mostly stimulated by the black chokeberry extract that may be conditioned by the other active substances available besides anthocyanins and their additional contribution to biological activity.

The study of MDA as an indicator of LPO showed that after the injection of CP, increased lipid peroxidation activity was observed, that is, decreasing protective antioxidant functions of the body. The injection of the anthocyanin standard mixture into both intact rats and rats affected by CP resulted in a reduction in LPO product accumulation.

It is evident that the antioxidant properties of anthocyanidins have an impact on oxidative stress processes caused by CP, resulting in reduced LPO product accumulation. The obtained results are consistent with previous studies that showed that anthocyanins caused statistically significant reductions in MDA levels in rat blood [37,38,39].

Reduction in free radical formation, reactive oxygen and nitrogen species and LPO products as affected by anthocyanidins and other plant phenols is shown by a number of scientists [40,41,42,43], and there are studies [40,41,42] showing that anti-inflammatory properties’ manifestation, directly dependent on the immune system activity, for plant extracts enriched with anthocyanidins and other phenols is associated with high antioxidant activity.

## 4. Materials and Methods

### 4.1. Sampling

As a raw material, we used the berries of the Black Pearl chokeberry bush. The chokeberry variety was confirmed by amplified fragment length polymorphism (AFLP) of the DNA fragments. The mature Aronia berries in umbel form were gathered in the Shujskie Yagodi farm (Ivanovo region) during the harvest season in September 2021. In summer 2021, the average temperatures in the Ivanovo region were within the range of +17–25 °C (daytime) and +9–15 °C (night). The berries had a sour-sweet and slightly puckery taste, purple-black color, gray coating, bright-red pulp and spherical form with a maximum diameter of 0.9 cm and an average mass of 1.2 g. The berries were gathered randomly from ten bushes, in an amount of 0.5 kg per plant. At the time of berry harvest, the chokeberry bush was 5 years old. The berries were washed with distilled water to remove dust and contaminants and dried with cotton cloth and stored in a freezing compartment at −35 °C for further research.

### 4.2. Aronia Extract Preparation

Since the work studied the antioxidative properties in correlation to the chemical composition, the first step was to obtain ethanol extract from the frozen berries. The frozen chokeberry berries were ground to a paste in a laboratory mill (LM 202, Russia) and extracted by maceration. Ground berries were transferred to flat-bottomed conical flasks pre-purged with inert argon and extracted with 70% ethanol solution at a 1:5 ratio of biomass to solvent and stirred at 500 rpm using an automatic magnetic stirrer (Ohaus, Guardian 7000) at the temperature of 45 °C for 1.5 h with a constant supply of dry argon to avoid oxidation of active compounds [44,45].

The homogenate obtained as a result of extraction was centrifuged at 13,500 rpm for 15 min to precipitate large particles and the suspension (Biosan SIA centrifuge, microspin 12). The extract was concentrated on a rotary evaporator (LabTex Re 100-Pro) over a water bath at a temperature of 30–32 °C and a pressure of 6.7 mbar until the solvent was completely removed. The dried extract was stored in a medical freezing compartment at –35 °C for further analysis. Further, the extract was used as a reference sample.

### 4.3. Anthocyanin Extract Preparation

Frozen and ground berries were extracted with 50% ethanol solution acidified with 1% hydrochloric acid (by volume) and then added to the material with a solvent ratio of 1:5. The prepared homogeneous sample was transferred to a flask and extracted over a water bath stirred at 500 rpm with an automatic magnetic stirrer (IKA RCT basic) at 45 °C for 1.5 h. The extracting procedure was repeated twice with the addition of a new portion of the material. The prepared extract was centrifuged to precipitate large particles and suspensions using a US 1536E centrifuge for 20 min at 6200× *g* and filtered using a Schott funnel with a glass filter with a porosity of 10–16 microns (Class 4) at a pressure of 20 mbar for additional purification and clarification, in accordance with [46].

The anthocyanin extract was purified using a polar ion-exchange resin NKA-9 (Shanghai Mosu Science Equipment Co., Shanghai, China) acting as an adsorber. The bottom of the 3 × 45 cm glass column was covered with a layer of calcined glass wool, a layer of adsorbing resin and 800 °C calcined silica sand (of particle distribution 0.3–1 mm); each adsorbing layer was 10–12 cm high, and calcined glass wool was included between the layers. In addition, a piece of glass wool was placed over the adsorbing layer to avoid damage to the latter. Afterward, the column was washed using 50% ethanol acidified with 1% HCl (by volume) and subsequent raw extract with constant pouring; the resulting extract was collected as separate portions of 10 mL until no staining was observed in the eluted extract. The resulting anthocyanin extract was combined as described in [19]. The pH of the prepared anthocyanin was 3.93.

### 4.4. Chemical Composition Analysis

The total anthocyanin content on cyanidin-3-O-glucoside equivalent (mg) basis per gram of dry extract was determined by the spectrophotometric differential method [47]. The optical density was measured at 520 and 700 nm using a spectrophotometer (LEKI SS1207).

The total sugar content was determined spectrophotometrically at 520 nm using a 5% aqueous solution of phenol and concentrated sulfuric acid [48]. As for the reference solution, this was prepared using xylose as a base. The total content of flavonoids was determined by the Stankovich method [49]. One milligram per milliliter concentration extracts in the amount of 1 mL and a 2% AlCl_3_ ethanol solution (1 mL) were dissolved in an appropriate solvent and diluted 50 times. The prepared samples were kept for one hour at an ambient temperature. The optical density was determined by spectrophotometer at λ_max_ = 415 nm. The same measurement was performed for the rutin solution used as a standard. The flavonoid content was determined on a rutin basis. The results were written as rutin equivalent milligrams per gram of dry extract (Rut mg/g). The analysis results are given in Table 1.

### 4.5. Chromatographic Analysis of Anthocyanin Extract

The collected anthocyanin extract was subsequently subjected to column chromatography to separate the anthocyanins and anthocyanidins present in *Aronia melanocarpa*. The glass chromatographic column was densely filled with silica gel with the particle size of 0.06–0.2 mm. Using column chromatography, 30 eluted extracts were collected using a solvent system of methanol:acetone (30:70) (I) and ethanol:acetone (55:45) (II). Using these mobile phases, 15 mL extracts were collected, and each portion was subjected to thin-layer chromatography on TLC silica gel 60 F_254_ aluminum plates. For stain visualization, we used a TCX 254/365 UV irradiator with a 254 nm wavelength. Chromatography over the plates in I and II systems showed the 6–9, 13–15, 20–22 and 26–27 extracts consisting of one component. The isolated fractions of individual anthocyanins were identified by ESI mass spectrometry and HPLC. A mass spectrometer with ion trap and ESI and atmospheric pressure chemical ionization (APCI), Amazon XBruker Daltonix GmbH (Bremen, Germany), was used (Table 3). Fractions 1–5, 10–12, 17–19 and 23–25 were mixtures. The retardation factor (R_f_) for these extracts was 0.63, 0.51, 0.45 and 0.23, respectively [50]. The collected extracts were concentrated to crystalline fines at 35–37 °C and a pressure of 0.10–0.13 mbar using a rotary evaporator. The obtained extracts were stored at +5 °C in vials in an argon medium. Based on analysis results, the major component of *Aronia melanocarpa* anthocyanin extract is cyanidin-3-O-galactoside (Table 2). For the purpose of this study, this extract was dissolved in the estimated amount of 1.3-propylene glycol to obtain a 1% solution.

Identification of individual compounds in the extract and fractions was carried out by HPLC using an Agilent 1260 Infinity II LC Multiple Wavelength Detector chromatograph (USA) with a multiwave spectrophotometric detector (Table 4). HPLC analyses were performed using Zorbax Eclipse C18 (4.6 × 150 mm) and Agilent Hiflex H (250 = 4.6 mm) columns. To detect flavonoids, elution was performed in a gradient way. As the mobile phase, we used the mixture of 0.1% formic acid and acetonitrile solution in the ratios 1:0, 1:9 and 9:1 and 0.1% formic acid and acetonitrile solution in the ratios 1:0, 1:9 and 9:1 for the flavonoids. The eluent velocity was 1.0 mL/min, the volume of the injected sample was 10 µL, the elution time was 30 min and the temperature of the column heating block was 45 °C [51].

Anthocyanins were determined by hydrophilic HPLC with a reversed-phase column; the elution was performed with a solution containing 30 vol.% acetonitrile and formic acid in distilled water. The temperature of the columns’ heating block was 40 °C; the feeding rate of the mobile phase was 1 mL/min. The injection volume was 15 µL [52].

### 4.6. Chemicals and Reagents

Cyanidin standards, namely cyanidin, purity ≥95.0%; Cy-3-Gal (cyanidin-3-O-galactoside), purity ≥95.0%; and Cy-3-Glu (cyanidin-3-O-glucoside), purity ≥98.0%, were acquired from PhytoLab GmbH & Co. KG, Germany (Merck KGaA, Darmstadt, Germany) (Figure S1). *Echinaceae purpurea* tincture acquired from GalenoPharm, Russia, was used as an immunomodulator. Following the described method of anthocyanin preparation, an anthocyanin-rich extract with an anthocyanin content of ≥91.7% was extracted from the berries of *A. melanocarpa*, and *A. melanocarpa* cyanidin-3-O-galactoside extract (Cy-3-Gal Aronia) with a purity of ≥93.7% was in turn extracted from it.

### 4.7. Antioxidant Activity and Biologically Active Substance Contents in Aronia Extracts

The antioxidant activity of Aronia berry extracts was studied by chemiluminescence and colorimetric methods to determine the ability of substances to interact with free peroxide radicals, namely by AAPH and DPPH, respectively. Reagents acquired from Sigma Aldrich, USA, were used for the AAPH method; reagents acquired from Alfa Aesar, USA, were used for the DPPH method.

The chemiluminescence AAPH assay is described in [53] and was adapted for the Lum-1200 luminometer (LLC DISoft, Moscow, Russia) [54]. The results were processed on a personal computer using the PowerGraph and OriginLab software. In the work of Lissi et al. [55], two approaches to measuring the total antioxidative capacity, taking into account this feature of the curves, are described—the TRAP method and the TAR method. It is believed that TRAP reflects the amount of antioxidants in the system, and TAR reflects antioxidant activity, i.e., the rate of the antioxidant interaction with radicals. The TRAP method is based on the measurement of the CL latency period. The TAR method was used to determine the value of CL intensity quenching.

The DPPH assay was performed according to the method of Brand-Williams et al. [56] with some modifications. The stock solution was prepared by dissolving 24 mg DPPH with 100 mL ethanol and then stored at −20 °C until needed. The working solution was obtained by mixing 10 mL stock solution with 45 mL ethanol to obtain an absorbance of 1.1 ± 0.02 units at 515 nm using the spectrophotometer. Berry extracts (200 µL) were allowed to react with 800 µL of the DPPH solution for 30 min in the dark. Then the absorbance was taken at 517 nm. Antiradical activity was defined as the amount of antioxidant needed for a 50% reduction in the initial concentration of DPPH (effective concentration = EC_50_ ((mol/L) AO/(mol/L) DPPH). The effectiveness of the antioxidant was defined as antiradical power (ARP), defined as 1/EC_50_. The higher the ARP, the more effective the antioxidant.

### 4.8. Rat Erythrocyte In Vitro Tests for Extracts’ Protective Activity on Osmotic and Peroxide Hemolysis Models

The membrane-stabilizing action of the studied compounds was evaluated using methods that initiate osmotic and peroxide damage to rat erythrocyte membranes [57] and are adapted for the BioTek Epoch microplate reader (Winooski, VT, USA) [58]. To simulate osmotic hemolysis, a hypotonic 0.3% sodium chloride solution was added to the suspension of red blood cells. To simulate free radicals, a Fenton reagent was used, represented by a mixture of 0.01 mg/mL heptahydrate iron (II) sulfate and 0.2 mg/mL hydrogen peroxide. The studied cyanidin samples were added to the model systems in the concentration range from 0.0015 to 0.1 mg/mL. The intensity of hemolysis was assessed against the change in the optical density of the supernatants at a 543 nm wavelength. The membrane-stabilizing effect of the extracts was evaluated as the percent of hemolysis inhibition (C) and calculated by the following Formula (1):

(1) $$C\left(\%\right)=\frac{\left(D_{1}-D_{0}\right)}{D_{0}}\times 100,$$

where C (%)—hemolysis inhibition, D_1_—optical density of the tested sample, D_0_—optical density of the reference sample.

### 4.9. In Vitro Tests on the Lymphocyte Cell Line RPMI-1788

The tests were carried out using a conditionally normal cell line of human B lymphocytes, namely RPMI–1788 lymphoblasts obtained from the Russian Collection of Cell Cultures of the D.I. Ivanovskiy Institute of Virology (Moscow, Russia). The cells were maintained at 37 °C in RPMI-1640 medium supplemented with 10% fetal bovine serum, 1% essential amino acids and gentamycin antibiotic [59]. Cells were grown at 37 °C with 5% CO_2_.

#### 4.9.1. Determination of Cytoprotective Properties In Vitro

A cell suspension of RPMI-1788 was prepared at a concentration of 10^5^ cells/mL. A 200 µL suspension was pipetted into a 96-well plate and incubated for 24 h. To determine the cytoprotective effect in compliance with [60], CP toxicant at a concentration of 1.25 mg/mL was added together with the test subjects. Neither extracts nor CP was added into the growth medium of the negative (reference) control during cell cultivation. Only CP was added into the growth medium of positive control cells at an equivalent concentration of 1.25 mg/mL without adding the test substances. The cells were again incubated for 24 h. For each group of cells, the cultivation was carried out in triplicate.

#### 4.9.2. Cell Color Staining and Counting

To assess the viability of cell culture, a full growth medium also containing fluorescent dyes was prepared, assuming 198 µL total growth medium, 2 µL Hoechst 33,342 (1 mg/mL concentration) and 0.5 µL propidium iodide per well. Further, the culture liquid was replaced with a prepared dye-containing growth medium and incubated for 45 min. After incubation, live and dead cells were counted on a Millipore (Darmstadt, Germany) Guava easyCyte flow cytometer.

### 4.10. In Vivo Tests

All the procedures were carried out under the ethical guidelines of Kazan (Volga Region) Federal University (protocol No. 4 dated 18 May 2017). The studies were carried out on Wistar laboratory male rats with induced immune function decrease (immunosuppression) due to cytostatic CP administration. Against the background of induced immunodeficiency in rats, we studied the influence of the Aronia extractives on the immune status of animals, namely the number of leukocytes and their subpopulation ratio in peripheral blood and the functional state of immune cells (chemiluminescent and phagocytic activity, cytokine production).

As a preventive measure, rats within 7 days were treated orally with water (Control Group or Group C), cyanidin-3-O-galactoside of *Aronia melanocarpa* extracts (Group A) and *Echinacea purpurea* herb tincture (Group E) at doses of 50 mg/kg on dry mass. The Cy-3-Gal and Cy-3-Glu standards were mixed at 15.65:1. The mixture was dosed in amount 33.3 mg/kg and was correspondingly equivalent to the cyanidin content in the cyanidin-3-O-galactoside extract of Aronia berries (Group G). CP was then injected once abdominally at a dose of 25 mg/kg, and its oral administration was continued for the next 7 days. The first blood sampling was performed on the 1st day, before the test had been started. The obtained results were accepted as reference values. After that, we tested the blood again on the 8th day, i.e., one day after CP administration, then on days 14 and 21, i.e., 7 and 14 days after administration of extracts with induced immunosuppression in the background.

#### 4.10.1. Hematological Studies

To determine the number and subpopulation of the immune cells in peripheral rat blood, a Mythic 18 Vet (Orphee SA, Plan-les-Ouates, Switzerland) automatic hematology analyzer and a special reagent kit were used. The rat blood was tested to determine the total counts of white blood cells (WBCs), lymphocyte count in absolute units and in relation to the total count of white blood cells (LYM and LYM%, respectively), monocytes (in a similar way, i.e., MON and MON%) and granulocytes (GRA and GRA%). Red blood cell (RBC) count, hemoglobin (HGB) concentration, platelet (PLT) count and the mean platelet volume (MPV) were determined.

#### 4.10.2. Functional State of Neutrophilic Granulocytes in Rats’ Blood

The neutrophil granulocyte population is interesting as it is one of the most active cell populations, among the first to respond to antigenic aggression and damaging factors.

Luminol-dependent chemiluminescence (CL) evaluations are based on neutrophils’ ability to dramatically alter the metabolic profile and thus demonstrate a higher oxygen consumption, activity of membrane-bound NADP/H-oxidase and release of ROS from cells. The analysis of CL neutrophils reveals the formation of ROS by cells, including superoxide anion, singlet oxygen, hydroxyl radical, hydrogen peroxide. Luminol, which becomes an oxygenate under the influence of oxygen metabolites generating an optical photon, was used to enhance CL, which significantly increases the reaction sensitivity.

To evaluate the formation of ROS by neutrophils, a recording was made of the own light emission of cells isolated from the peripheral blood of experimental rats using the method described in [61], without the use of a CL activator (spontaneous activity) as well as a response signal of neutrophils’ CL to activation by zymosan (induced activity).

For the kinetic curve of the luminol-dependent CL of neutrophils, the following parameters were determined: the time showing peak CL (T_max,_ s), maximum luminous intensity (I_max_, c.u.) and area under the CL curve (AUC, c.u.) for spontaneous CL (AUC_1_) and CL induced by zymosan (AUC_2_).

The amplification of the induced CL vs. spontaneous CL was determined by the area ratio under the AUC_2_/AUC_1_ chemiluminescence curve, thus determining the I_act_, i.e., the activation index (c.u.).

The evaluation of phagocytosis, or bactericidal activity, was carried out using cytofluorometry employing the Millipore (Germany) Guava easyCyte flow cytometer. Inactivated bacteria *E. coli* labeled with fluorescein-5-isothiocyanate (FITC) (Sigma Aldrich, Germany) were used as an agent for phagocytosis using the method described in [62,63,64]. The phagocytosis reaction was performed by incubation of the leukoconcentrate (100 µL), obtained by the method described in [61] and containing 1 × 10^6^ cells per 1 mL with a suspension of FITC-labelled bacteria *E. coli* (10 µL, the number of bacteria being 5 × 10^7^ per 1 mL), for 30 and 120 min at 37 °C.

The phagocyte number (PN) of neutrophils for FITC-labelled *E. coli* was calculated as the number of bacterial particles phagocytosed per neutrophil or monocyte; phagocytic activity (PA) was calculated as the number of active phagocyte-absorbed FITC-labelled bacteria to the total number of monocytes or neutrophils; phagocyte index (PI) was calculated as the bacteria average for any neutrophil or monocyte; and phagocytosis completion index (PCI) in c.u. was defined as the ratio of PN in 30 min to PN in 120 min [63,65].

#### 4.10.3. Lipid Peroxidation in Rats’ Blood

The extent of LPO was determined by the MDA content in erythrocytes of rat blood. MDA in blood was determined by carrying out the reaction between the reactive agent and thiobarbituric acid as described by Buege and Aust [66], but with a slight modification of the method. Red blood cells in the amount of 0.1 g washed with normal saline were homogenized into 0.15 mol L^−1^ KCl at a ratio of 1:9 with the help of a glass homogenizer. One volume of red blood cells was thoroughly mixed with two volumes of a matrix solution of 15% wt./vol. trichloroacetic acid, 0.375% wt./vol. thiobarbituric acid and 0.25 mol × L^−1^ hydrochloric acid. The solution was heated for 15 min over a boiling water bath. After cooling, the sediment was centrifugated at 1000× *g* for 10 min. The absorption of the transparent supernatant was determined at 535 nm, and the MDA concentration was calculated using 1.56 × 10^5^ mol^−1^ cm^−1^ as the molar absorption coefficient. The MDA results were expressed in nanomoles per gram of blood with initial moisture.

### 4.11. Statistical Analysis

The data were processed using Microsoft Excel 2016 and OriginPro 9.5 (OriginLab Co., Northampton, MA, USA). Data were compared using a non-parametric Kruskal–Wallis test. The precise *p*-values were calculated for the pairwise comparisons between the groups using the Mann–Whitney test. SPSS 23.0 statistical software (Chicago, IL, USA) was employed for data analysis. The obtained values were represented as mean values ± the standard deviation of the mean. The level of *p* < 0.05 was considered statistically significant.

## 5. Conclusions

In this study, a highly purified anthocyanin fraction of *Aronia melanocarpa* berries was extracted, and its composition was analyzed by HPLC and spectrophotometry methods. It was found that the major component of the anthocyanin fraction was cyanidin-3-O-galactoside, constituting 73.5% of the dry extract mass.

According to the AAPH test, the total antioxidant capacity of the tested substances is reduced in the following series: rutin, Cy-3-Gal Aronia, Cy-3-Gal standard and Cy-3-Glu standard. The latent period is reduced in the following series: rutin, Cy-3-Glu standard Cy-3-Gal standard and Cy-3-Gal Aronia. According to the DPPH test, Cy-3-Gal Aronia exhibits the highest antioxidative activity among the anthocyanins studied, at the same time exhibiting activity inferior to that of rutin.

Cyanidins were found to exhibit protective activity on the erythrocyte membrane, protecting cells from the damaging action of the hypotonic medium and reactive oxygen species generated by the Fenton reaction, with Cy-3-Gal Aronia being the most effective. It has been shown that the Cy-3-Glu and Cy-3-Gal standards exhibit cytoprotective activity, resulting in increased vitality of the RPMI-1788 cell line under the toxic effects of cyclophosphamide.

It has been shown that oral administration of Cy-3-Gal Aronia berries, as well as a mixture of Cy-3-Glu and Cy-3-Gal standards in an equivalent ratio, leads to the manifestation of an immunostimulating effect, significantly superior to that of Echinacea tincture.

Cy-3-Gal of Aronia berries is responsible for a decrease in the level of lipid peroxidation products (malondialdehyde) in the blood both in a normal physiological status and in cases of immunosuppression induced by cyclophosphamide.

Studies have indicated numerous benefits associated with chokeberry consumption included in a daily diet. The pro-health potential of black chokeberry fruit and its products indicates that all fractions could be consumed as a source of antioxidants and valuable nutrients with potential applications in the food and pharmaceutical industry, including as bioactive functional ingredients—targets for combating factors of a weakened immune system.

## Figures and Tables

**Table 1:** Total content of sugars and natural antioxidants in ethanol extract and cyanidin-3-O-galactoside extract of *Aronia melanocarpa* berries (Cy-3-Gal Aronia) (on dry basis).

Materials	pH	Total Sugars ^1^, mg Xy/g Extract	Total Flavonoids ^2^, mg Rut/g Extract	Total Anthocyanins, mg/g Extract
Aronia ethanol extract	7.27	63.65	37.50	93.60
Cy-3-Gal Aronia	3.93	0.125	15.35	917.31

^1^ Total sugar content per xylose equivalent (Xy); ^2^ total flavonoids per rutin (Rut) equivalent.

**Table 2:** Anthocyanin quantities identified in extract and cyanidin-3-O-galactoside fraction of *Aronia melanocarpa* berries.

Sample	Cyanidin-3-O-galactoside	Cyanidin-3-O-glucoside	Cyanidin-3-O-arabinozide	Cyanidin-3-O-xyloside
	mg/g ext.			
Aronia ethanol extract	58.97	6.21	18.15	8.89
Cy-3-Gal Aronia	735.33	12.58	115.22	31.33

**Table 3:** Column chromatography output of individual anthocyanin fractions.

Fraction	Output Relative to Anthocyanin Fraction	Major Molecular Mass Peak, [M-H]		Anthocyanins			
				Cyanidin-3-O-galactoside	Cyanidin-3-O-glucoside	Cyanidin-3-O-arabinozide	Cyanidin-3-O-xyloside
	%	Anth.	Anth.HCl^−^	%	%	%	%
6–9	81.7	447	484	93.7	0.15	2.1	0.49
13–15	3.1	447	484	1.1	97.8	0.7	0.3
20–22	11.3	417	453	0.6	0.3	94.9	0.4
26–2	3.5	417	453	0.5	0.2	3.7	93.9

**Table 4:** Comparative flavonol contents in the extract and anthocyanin fraction.

Sample	Individual Form, mg/g Dry ext.					Glycosidic Form, mg/g Dry ext.					
	Quercetin	Kaempfe-rol	Rhamnetin	Isorhamnetin	Dihydroquercetin	Quercetin-3-O-rutinoside	Hesperetin 7-O-rutinoside	Quercetin-3-O-rhamnoside	Quercetin-3-O-galactoside	Quercetin-3-O-glucoside	Dihydroquercetin hexoside
Aronia ethanol extract	12.35	0.11	0.63	-	2.17	15.35	0.97	0.93	0.18	0.49	0.27
Cy-3-Gal Aronia	3.235	0.029	0.371	0.023	1.153	7.973	0.69	0.067	0.036	0.075	0.115

**Table 5:** Antioxidant activity by DPPH assay of individual anthocyanins and anthocyanins extracted from *A. melanocarpa*.

Sample	EC_50_^1^, mg/mL	ARP ^2^, mg/mL^−1^
Cyanidin-3-O-galactoside	0.221	4.534
Cyanidin-3-O-glucoside	0.169	5.909
Cy-3-Gal Aronia	0.154	6.484
Quercetin-3-O-rutinoside (Rutin)	0.089	11.170

^1^ EC_50_—effective concentration for 50% reduction in the initial concentration of DPPH (2.2-diphenyl-1-picrylhydrazyl). ^2^ ARP— antiradical power defined as 1/EC_50_.

## Data Availability

Not applicable.

## Supplementary Materials

The following supporting information are available online at: https://www.mdpi.com/article/10.3390/plants11233333/s1, Figure S1: HPLC chromatograms of standard compounds. (a) Cyanidin-3-O-galactoside, retention time 23.27 min; (b) cyanidin-3-O-glucoside, retention time 24.35 min; (c)—cyanidin-3-O-arabinozide, retention time 25.79 min; (d) cyanidin-3-O-xyloside, retention time 33.17 min; Figure S2: HPLC chromatograms of anthocyanin fractions of chokeberry. (a) Fractions 6-9, (b) fractions 13-15, (c) fractions 20-22, (d) fractions 26-27; Figure S3: Electrospray ionization (ESI) mass spectra of anthocyanin fractions.
