# Supplementary figures and images for: Antioxidative and Immunomodulating Properties of Aronia melanocarpa Extract Rich in Anthocyanins
Source: Plants (Basel). 2022 Dec 1;11(23):3333. doi: 10.3390/plants11233333 (PMC9737032; doi:10.3390/plants11233333)

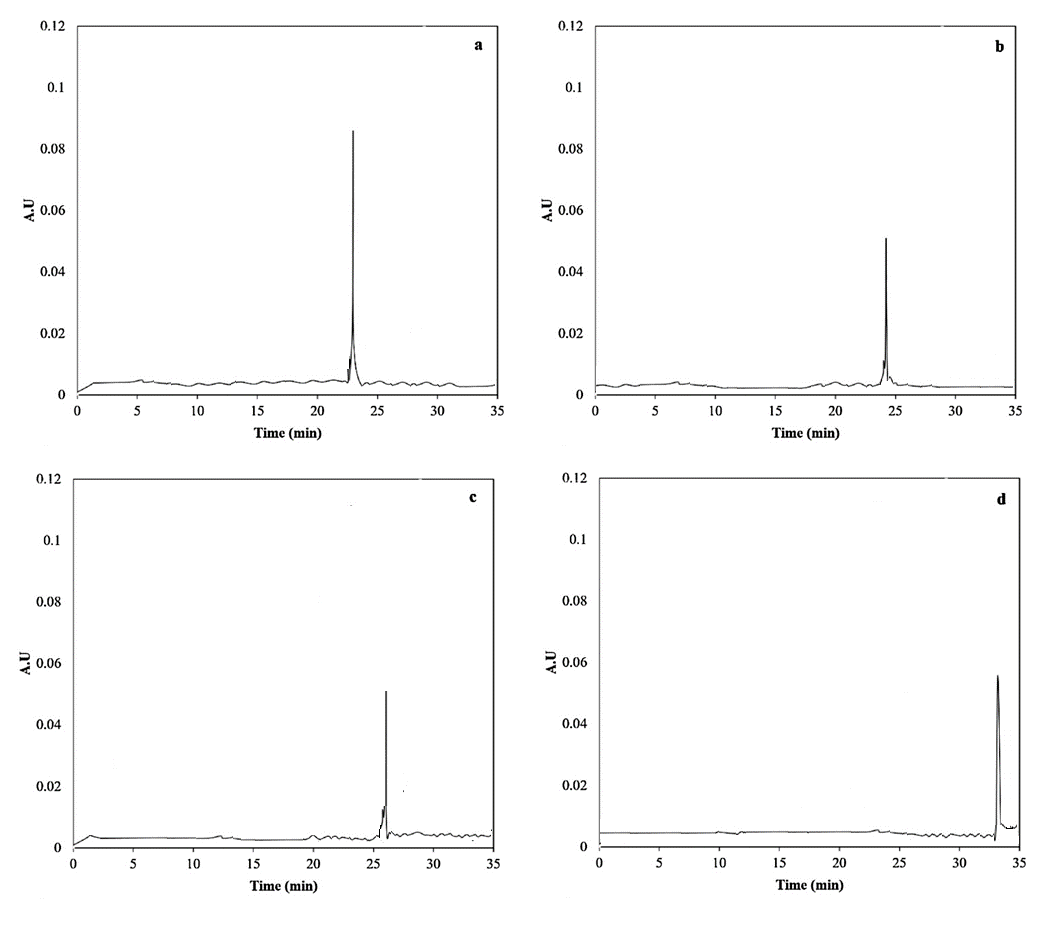

Supplement: Supplementary file 1 [file plants-11-03333-s001.zip › plants-2013021-supplementary/Fig.S1 Supplementary material.bmp]

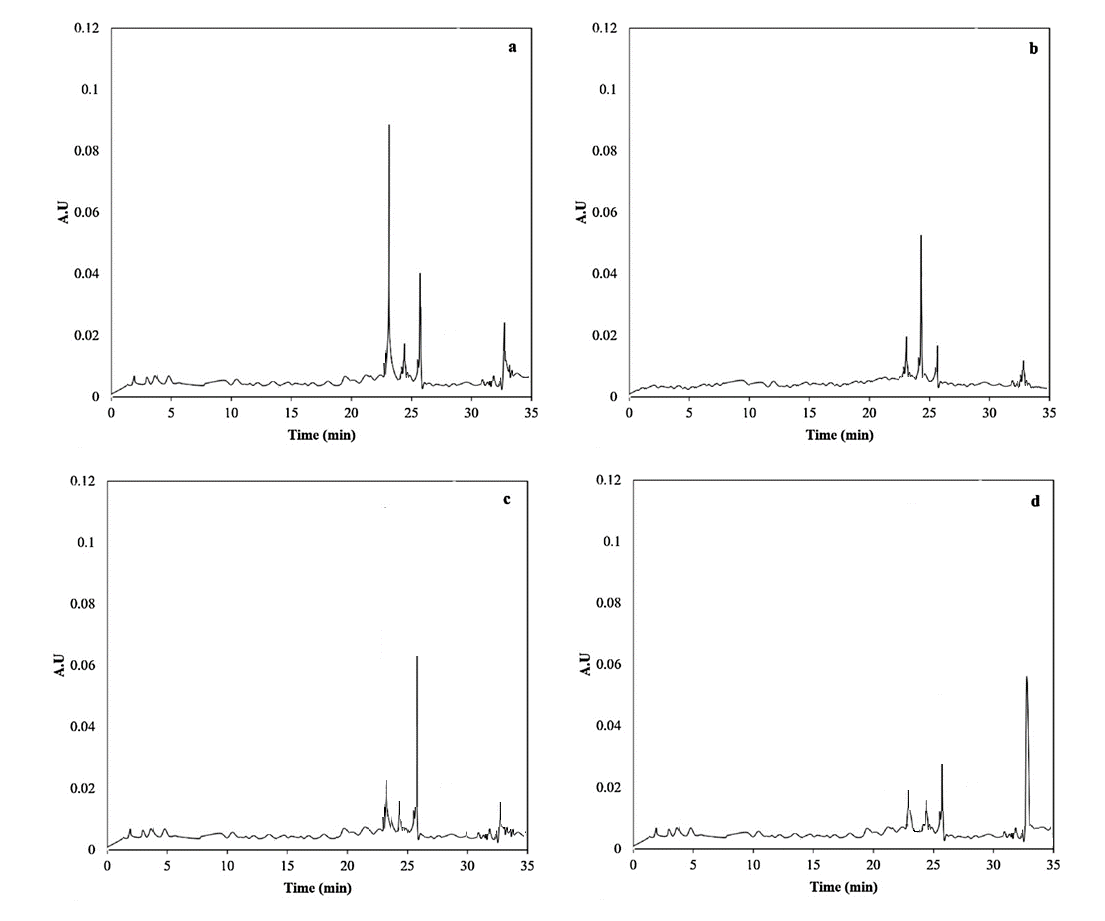

Supplement: Supplementary file 1 [file plants-11-03333-s001.zip › plants-2013021-supplementary/Fig.S2 Supplementary material.bmp]

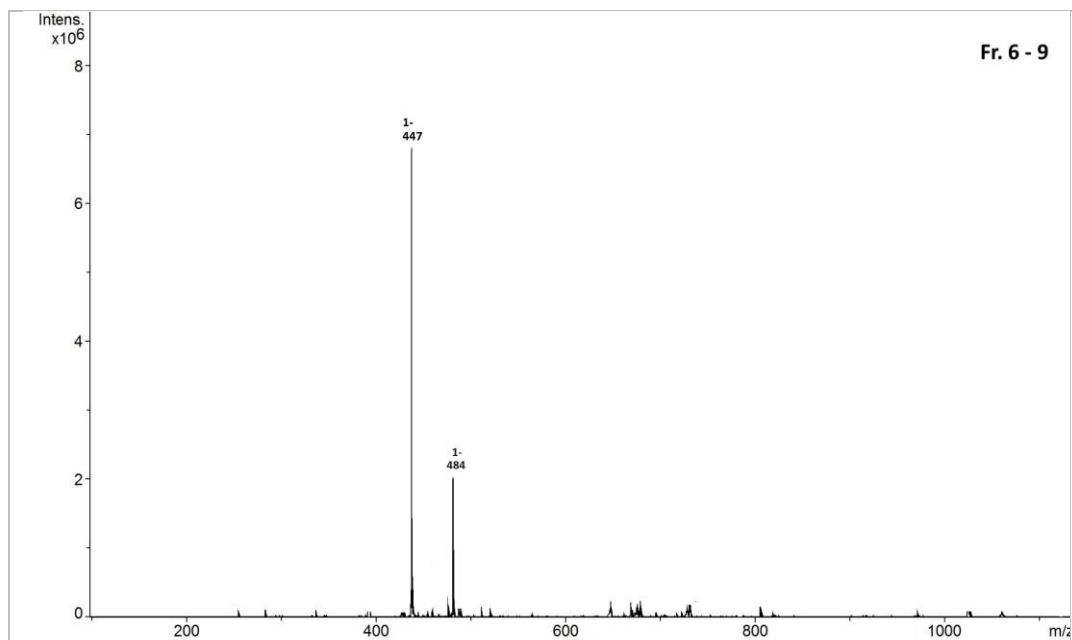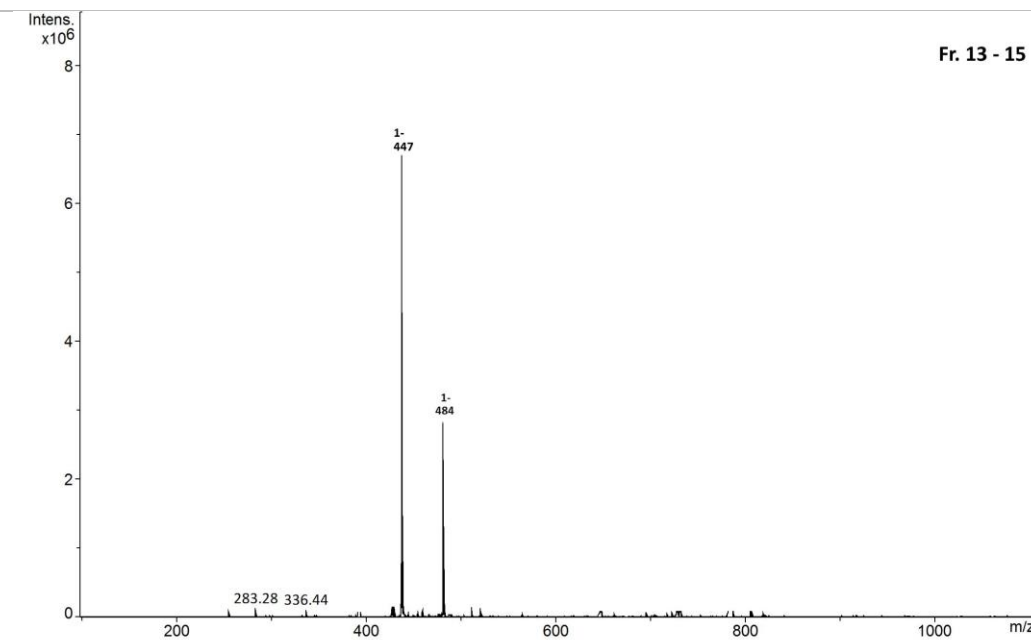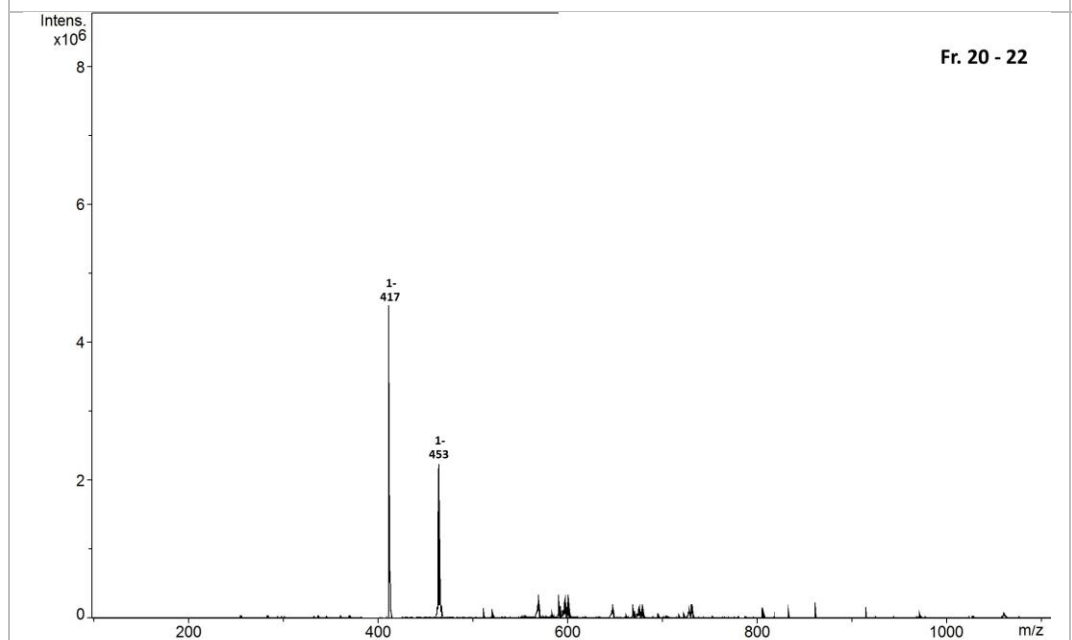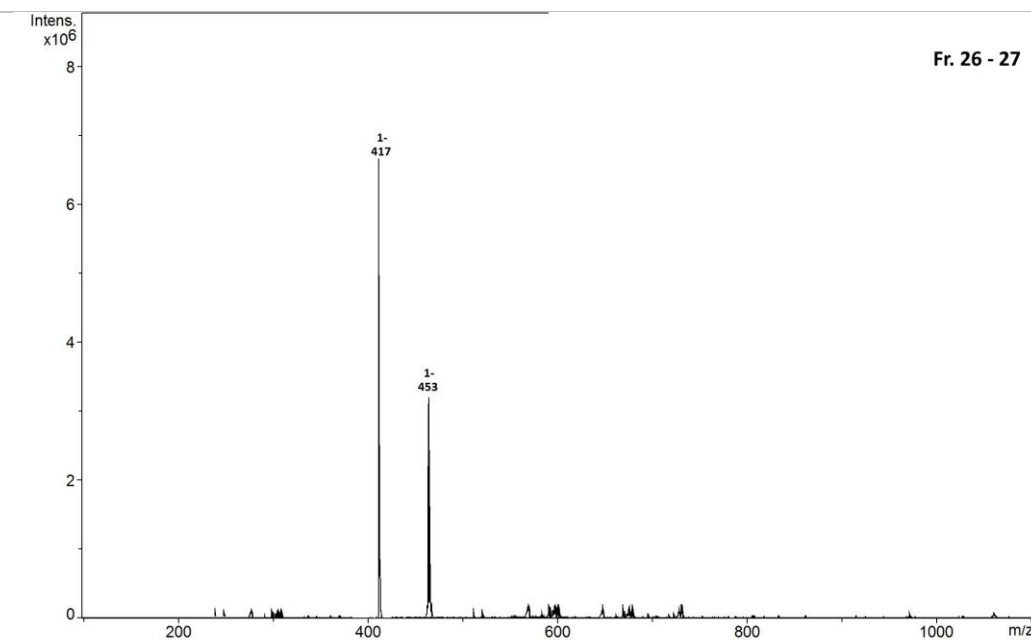

Supplement: Supplementary file 1 [file plants-11-03333-s001.zip › plants-2013021-supplementary/Fig.S3 Supplementary material.pdf]
